# Comparative Angiogenic Activities of Induced Pluripotent Stem Cells Derived from Young and Old Mice

**DOI:** 10.1371/journal.pone.0039562

**Published:** 2012-06-27

**Authors:** Hirohiko Suzuki, Rei Shibata, Tetsutaro Kito, Takashi Yamamoto, Masakazu Ishii, Naomi Nishio, Sachiko Ito, Ken-ichi Isobe, Toyoaki Murohara

**Affiliations:** 1 Department of Cardiology, Nagoya University Graduate School of Medicine, Nagoya, Aichi, Japan; 2 Department of Immunology, Nagoya University Graduate School of Medicine, Nagoya, Aichi, Japan; Northwestern University, United States of America

## Abstract

Advanced age is associated with decreased stem cell activity. However, the effect of aging on the differentiation capacity of induced pluripotent stem (iPS) cells into cardiovascular cells has not been fully clarified. We investigated whether iPS cells derived from young and old mice are equally capable of differentiating into vascular progenitor cells, and whether these cells regulate vascular responses *in vivo*. iPS cells from mouse embryonic fibroblasts (young) or 21 month-old mouse bone marrow (old) were used. Fetal liver kinase-1 positive (Flk-1^+^) cells, as a vascular progenitor marker, were induced after 3 to 4 days of culture from iPS cells derived from young and old mice. These Flk-1^+^ cells were sorted and shown to differentiate into VE-cadherin^+^ endothelial cells and α-SMA^+^ smooth muscle cells. Tube-like formation was also successfully induced in both young and old murine Flk-1^+^ cells. Next, hindlimb ischemia was surgically induced, and purified Flk-1^+^ cells were directly injected into ischemic hindlimbs of nude mice. Revascularization of the ischemic hindlimb was significantly accelerated in mice transplanted with Flk-1^+^ cells derived from iPS cells from either young or old mice, as compared to control mice as evaluated by laser Doppler blood flowmetry. The degree of revascularization was similar in the two groups of ischemic mice injected with iPS cell-derived Flk-1^+^ cells from young or old mice. Transplantation of Flk-1^+^ cells from both young and old murine iPS cells also increased the expression of VEGF, HGF and IGF mRNA in ischemic tissue as compared to controls. iPS cell-derived Flk-1^+^ cells differentiated into vascular progenitor cells, and regulated angiogenic vascular responses both *in vitro* and *in vivo*. These properties of iPS cells derived from old mice are essentially the same as those of iPS cells from young mice, suggesting the functionality of generated iPS cells themselves to be unaffected by aging.

## Introduction

Therapeutic angiogenesis is a novel strategy for treating patients with severe peripheral arterial disease (PAD), lacking other options. The aim is to promote angiogenesis. We have performed therapeutic angiogenesis using autologous bone marrow mononuclear cell implantation into the ischemic muscles of patients with critical limb ischemia (TACT protocol) [1]. However, elderly patients with very severe PAD undergoing chronic hemodialysis or with uncontrolled diabetes had poor responses to the TACT procedure [2]. Recent clinical trials of stem and progenitor cell treatment have also been disappointing in subjects of advanced age, although safety has been confirmed [3], [4], [5], [6]. In this regard, advanced age is associated with decreased stem cell activities, which might diminish the capacity for tissue regeneration [7]. Therefore, it is necessary to assess the changes that occur in cells during aging, when considering the ultimate success of these strategies.

Novel embryonic stem (ES) cell-like pluripotent stem cells, or “induced pluripotent stem” (iPS) cells, were generated from mouse skin fibroblasts by introduction of four transcriptional factors [8]. iPS cells could be used repetitively and were capable of differentiating into various types of cells as needed. It was reported that various cardiovascular cells were directionally induced from mouse and human iPS cell-derived fetal liver kinase-1 positive (Flk-1^+^) cells *in vitro* [9], [10]. Recently, we demonstrated direct local implantation of mouse iPS cell-derived Flk-1^+^ cells to augment ischemia-induced angiogenesis in a mouse model [11]. Thus, iPS cells might be applicable to therapeutic angiogenesis. In the clinical setting, to utilize iPS cells for therapeutic angiogenesis, it is necessary to establish iPS cells from elderly patients. Very recently, we successfully established iPS cells from 21-month-old mice using bone marrow-derived myeloid cells [12]. However, the effect of aging on the differentiation capacity of iPS cells into cardiovascular cells has not been clarified. In the present study, we investigated whether iPS cells derived from young or old mice had similar capacities to differentiate into vascular progenitor and mature cells, and whether these progenitor cells can regulate the angiogenic process *in vivo*. We employed a murine hindlimb model of ischemia-induced angiogenesis. Our observations indicate iPS cell-derived Flk-1^+^ cells to differentiate into mature vascular cells, and to regulate angiogenic vascular responses.

## Materials and Methods

### Materials

Allophycocyanin (APC) Biotin conjugated anti-mouse Flk-1, Anti-Mouse CD144 (VE-cadherin) Biotin, Streptavidin Phycoerythrin (PE) and Streptavidin Fluorescein isothiocyanate (FITC) were purchased from eBioscience (San Diego, CA, USA). APC streptavidin was purchased from BD Pharmingen (San Diego, CA, USA). Streptavidin microbeads were purchased from Miltenyi Biotec (Bergisch Gladbach, Germany). Monoclonal anti-mouse α-smooth muscle actin (α-SMA) antibody and PKH26 Red Fluorescent Cell Linker Kit were purchased from SIGMA-ALDRICH (St Louis, MO, USA). Alexa Fluor® 555 conjugated goat anti-mouse IgG was purchased from Molecular probes (Invitrogen, Carlsbad, CA, USA).

### Cell Culture

A mouse iPS cell line, MEF (iPS cells derived from young mice), was generated from C57/BL6 mouse embryonic fibroblasts by introducing four factors (Oct3/4, Sox2, Klf4 and the c-Myc mutant c-Myc(T58A)) using retroviral vectors in our laboratory [12]. A mouse iPS cell line, BM21 (iPS cells derived from old mice), was generated from dendritic cells of 21-month-old C57/BL6 mice by introducing the four aforementioned factors using retroviral vectors. These iPS cells harbor the enhanced green fluorescent protein (EGFP) downstream from the CAG promoter. iPS cells were maintained in Dulbecco’s modified Eagle’s medium (Invitrogen) containing 10% Knockout Serum Replacement, 1% fetal bovine serum (FBS), nonessential amino acids, 5.5 mmol/L 2-mercaptoethanol, 50 U/mL penicillin, and 50 mg/mL streptomycin on feeder layers of mytomycin-C–treated mouse embryonic fibroblast cells stably releasing leukemia inhibitory factor (LIF). Cell differentiation was induced as described previously [9]. In brief, differentiation medium (DM) (α-minimum essential medium supplemented with 10% FBS and 5×10^−5^ mol/L 2-mercaptoethanol) was used for iPS cell differentiation. Fetal liver kinase-1 positive (Flk-1^+^) mesodermal cells were induced by cultivating iPS cells (plated at 1.7×10^3^ cells/cm^2^) in DM in the absence of LIF on type IV collagen-coated dishes (ASAHI GLASS CO., LTD, Tokyo, Japan).

Cultured cells were harvested after induction of undifferentiated iPS cells cultivated in DM on type IV collagen-coated dishes. Induced cells were stained with biotin conjugated anti-mouse Flk-1 antibody followed by APC streptavidin secondary antibody. Flk-1^+^ cells were incubated with streptavidin microbeads, and then sorted with a magnetic cell separation system (MACS). Purity was confirmed by flow cytometric analysis performed using the fluorescence-activated-cell sorter (FACS) instrument (BD FACS Canto, Becton Dickinson, NJ, USA) and Cell Quest software (BD Biosciences).

Purified Flk-1^+^ cells were also re-plated on type IV collagen-coated dishes. Five to seven days later, re-plated Flk-1^+^ cells had differentiated into mature vascular cells. Differentiated cells were assessed by staining with VE-cadherin or α-SMA.

The formation of vascular-like structures by iPS-derived Flk-1^+^ cells on growth factor-reduced Matrigel (BD Biosciences) was induced according to the manufacturer’s instructions. iPS-derived Flk-1^+^ cells were seeded onto Matrigel coated plates at 3×10^4^ cells/cm^2^ in EBM-2 medium containing EGM-2 (LONZA, Basel, Switzerland), and incubated at 37°C for 24 h. Network formation was assessed using an inverted phase contrast microscope (Nikon, Tokyo, Japan). The degree of tube formation was quantified by measuring the length of tubes in five randomly low power fields. In some experiments, incorporation of iPS-derived Flk-1^+^ cells was assessed by seeding human umbilical vein endothelial cells (HUVEC) and PKH26 Red Fluorescent Cell Linker Kit- or GFP-labeled iPS-derived Flk-1^+^ cells at a ratio of 1∶1 on Matrigel. Network formation was assessed using a fluorescence microscope to assess the frequency of labeled cell incorporation.

For detection of cell senescence, we stained for senescence-associated β-galactosidase (SA β-Gal) using a Senescence Detection kit (Bio Vision, Mountain View, CA, USA) according to the manufacturer’s instructions. Briefly, cells of interest were grown to 80% confluence in culture dishes. Cells were washed twice with phosphate buffered saline (PBS) and then fixed with 4% paraformaldehyde. Fixed cells were cultured with staining solution in an incubator (37°C, 5% CO_2_) for 24 hours. Stained cells were stored in 70% glycerol and observed under a microscope. Blue, especially around the nuclear area, indicated senescent cells.

**Figure 1:**
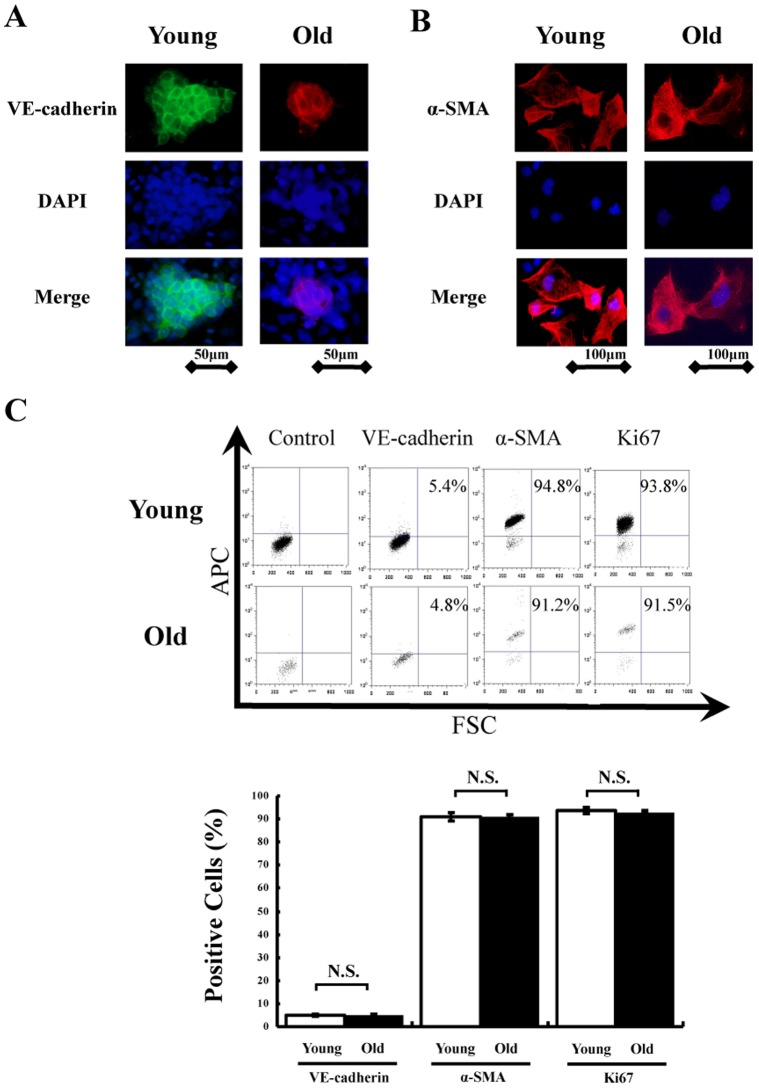
Differentiation into mature vascular cells *in vitro*. Sorted Flk-1^+^ cells derived from young and old iPS cells successfully differentiated into (A) mature endothelial cells (VE-cadherin positive) and (B) smooth muscle cells (α-SMA positive) 5 to 7 days after re-culture *in vitro*. Total nuclei were identified by DAPI counterstaining (blue). (C) Representative images of FACS analysis in differentiated cells (upper). FACS analysis was performed 5 to 7 days after re-plating of sorted Flk-1^+^ cells derived from young and old iPS cells on type IV collagen-coated dishes. Quantitative analysis of α-SMA, VE-cadherin and Ki-67 positive cells in differentiated cells (n = 5 in each group) (lower).

### Mouse Model of Hindlimb Ischemia and Cell Transplantation

Male KSN athymic nude mice were used for this study. Study protocols were approved by the Institutional Animal Care and Use Committee of Nagoya University School of Medicine. Mice, ages 8 to 12 weeks or ages 18 to 20 weeks, were subjected to operative unilateral hindlimb ischemia under anesthesia with sodium pentobarbital (50 mg/kg i.p.) as described previously [13]. Flk-1^+^ cells (2×10^5^ cells/mouse) or PBS as a control were injected into six different sites of adductor muscles in the ischemic limb after surgery. In other experiments, sorted Flk1^+^ cells were labeled with a PKH26 Red Fluorescent Cell Linker Kit, and then injected into ischemic adductor muscles.

We measured the ratio of the ischemic to normal hindlimb blood flow using laser Doppler blood flowmetry (LDBF) (Moor LDI, Moor Instrument Inc., Devon, UK) as described previously [13], [14], [15]. To minimize variations due to ambient light, blood flow was expressed as the ischemic to normal hindlimb LDBF ratio.

Capillary density in adductor muscles was analyzed to obtain specific evidence of vascularity at the microcirculatory level [14]. Tissue samples were obtained from the ischemic thigh adductor skeletal muscles on postoperative day 28. Tissue slices (8 µm in thickness) were prepared and stained with VE-cadherin followed by treatment with streptavidin PE or FITC-conjugated secondary antibody to detect VE-cadherin. The signals were detected and analyzed by fluorescence microscopy. Fifteen randomly selected microscopic fields from 2 different sections in each tissue block were examined for the presence of capillary endothelial cells, and the capillary muscle fiber ratio was expressed as the ratio of the number of capillaries to the number of muscle fibers per high-power field (×400).

**Figure 2:**
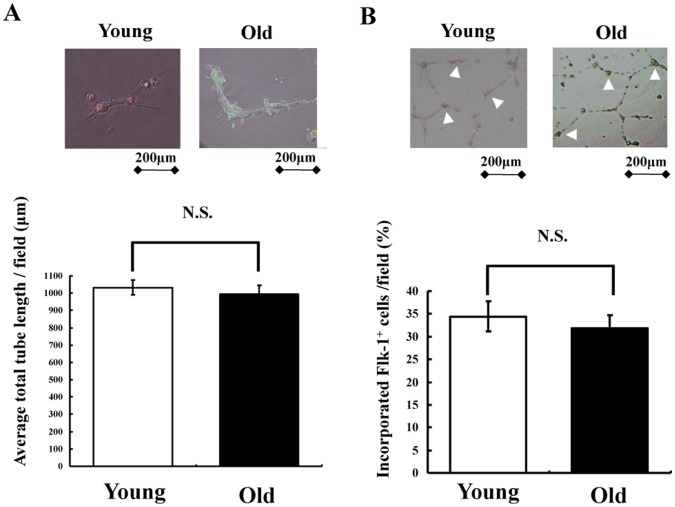
3D culture of sorted Flk-1^+^ cells *in vitro*. (A) Representative images of tube formation assay *in vitro* (upper). Sorted Flk-1^+^ cells derived from young and old iPS cells were cultured alone for 24 hours on Matrigel. Quantitative analysis of network projections formed on Matrigel for each experimental group (lower) (n = 3 in each group). (B) Representative images of HUVEC co-cultured with Flk-1^+^ cells (upper). Sorted Flk-1^+^ cells derived from young and old iPS cells were co-cultured with HUVEC for 24 hours on Matrigel. Flk-1^+^ cells derived from young and old iPS cells (white arrow head) were confirmed. The bar indicates 200 µm. Quantitative analysis of the number of Flk-1^+^ cells derived from young and old iPS cells into HUVEC on Matrigel (lower) (n = 3 in each group).

Total RNA from adductor muscles was extracted using the FastRNA Pro Green Kit. (MP Biomedicals, OH, USA). Reverse transcription was performed with 1 µg of RNA, random primers and MMLV reverse transcriptase (ReverTraAce-α TOYOBO, Osaka, Japan). Quantitative real-time PCR was performed with the LightCyclerT System (Roche Diagnostics, IN, USA) and QuantiTect SYBR Green PCR kit. Primers: mouse SIRT; sense 5′-AGTTCCAGCCGTCTCTGTGT-3′ and antisense 5′- CTCCACGAACAGCTTCACAA -3′, mouse ARF; sense 5′- ATCTGGAGCAGCATGGARTC -3′ and antisense 5′- CGAATCTGCACCGTAGTTGA -3′, mouse p21; sense 5′- GTACTTCCTCTGCCCTGCTG -3′ and antisense 5′- CAGAAGACCAATCTGCGCTT -3′, mouse VEGF; sense 5′-CAGGCTGCTGTAACGATGAA-3′ and antisense 5′- GCATTCACATCTGCTGTGCT-3′, mouse HGF; sense 5′- GGCAGCTATAAAGGGACGGTA-3′ and antisense 5′- CTTCTTCCCCTCGAGGATTT-3′, mouse IGF; sense 5′- CTACCAAAATGACCGCACCT-3′ and antisense 5′- CACGAACTGAAGAGCATCCA-3′, and mouse GAPDH; sense, 5′-AACTTTGGCATTGTGGAAGG -3′ and antisense, 5′-ACACATTGGGGGTAGGAACA -3′.

### Statistical Analysis

All data were obtained from at least three independent experiments. Student t test for comparison between two groups was performed. One-way ANOVA test for comparison among multiple groups was performed. Repeated-measures ANOVA test was used for the blood flow data analyses. All analyses were performed using PASW Statistics18 software (SPSS Inc, IL, USA). P<0.05 was considered significant. All data are shown as means ± S.E.

**Figure 3:**
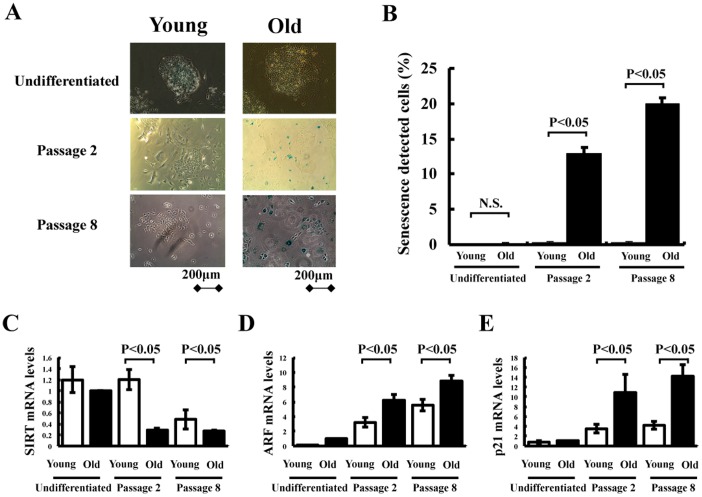
Senescence assay *in vitro*. (A) Undifferentiated and differentiated iPS cells were stained with a senescence detection kit to detect senescence associated-β-galactosidase (SA-β-Gal) around the nuclear area. (B) Quantitative analysis of the number of SA-β-Gal positive cells in undifferentiated and differentiated iPS cells. Expression of (C) SIRT and senescence associated genes such as (D) ARF and (E) p21 in Flk-1^+^ cells from young and old murine iPS cells determined by real-time PCR. SIRT, ARF and p21 mRNA levels were expressed relative to GAPDH mRNA levels (n = 3 in each group).

## Results

### Differentiation of Flk-1^+^ Cells Derived from iPS Cells from Young and old Mice into Vascular Cells

Undifferentiated iPS cells from young and old mice were cultured on collagen IV-coated dishes with DM as described previously [9]. We assessed the time course of Flk-1^+^ cell appearance by FACS analysis. Flk-1^+^ cells were induced from iPS cells, derived from young and old mice, after 3.5 days of culture, and peaked at day 7.5 (Figure S1A). The time courses of iPS cells from old mice were comparable to those of iPS cells from young mice. Based on these findings, we sorted Flk-1^+^ cells derived from iPS cells from young and old mice by MACS at day 7.5 of differentiation. FACS analysis of MACS-sorted positive cells showed more than 90% of these cells to be positive for Flk-1 (Figure S1B).

We induced mature endothelial cells and smooth muscle cells from Flk1^+^ cells. Sorted Flk1^+^ cells were re-cultured on type IV collagen–coated dishes and cultivated for 5 to 7 days with medium containing 10% FCS. Immunofluorescence analysis revealed that VE-cadherin^+^ endothelial cells and α-SMA^+^ smooth muscle cells were selectively induced from Flk1^+^ cells derived from iPS cells obtained from both young and old mice (Figure 1A and B). We also assessed the expressions of VE-cadherin, α-SMA and Ki67 by FACS analysis. Representative data of FACS analysis are shown in Figure 1C. Quantitative analyses revealed that mature vascular cells from young and old murine iPS cells accounted for 90% of α-SMA positive smooth muscle cells and 4–6% of VE-cadherin positive endothelial cells (Figure 1C). FACS analysis also showed nearly all of these populations in both young and old murine iPS cells to be positive for a proliferation marker, Ki67 (Figure 1C).

**Figure 4:**
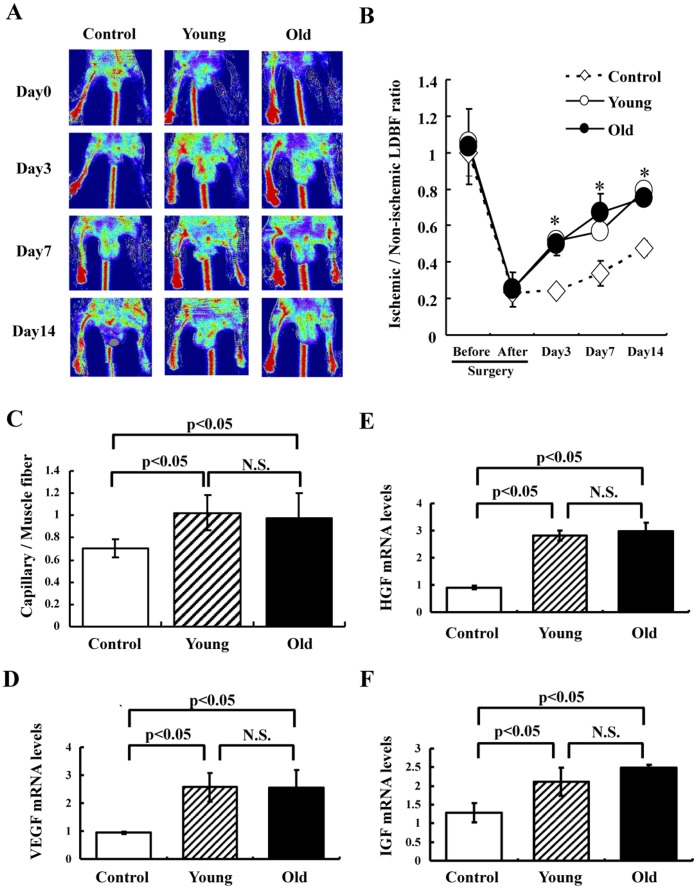
Effects of cell transplantation on blood flow recovery in the ischemic hindlimb. (A) Representative LDBF images. A low perfusion signal (dark blue) was observed in the ischemic left hindlimb of control mice (PBS), whereas high perfusion signals (white to red) were detected in the ischemic left hindlimb of mice transplanted with Flk-1^+^ cells derived from young and old mice (2×10^5^ cells) on postoperative days 3, 7 and 14. (B) Quantitative analysis of the ischemic to non-ischemic limb LDBF ratio on pre- (Day-1) and postoperative days 0, 3, 7 and 14 (Control: n = 8, Young: n = 4, Old: n = 4). *p<0.05 for mice injected with Flk1^+^ cells (2×10^5^) vs. control mice. (C) Capillary density analysis. Capillary density was determined at day 21 after surgery. Collected ischemic hindlimb muscle was stained with VE-cadherin. Capillary density was calculated as below. The number of VE-cadherin positive cells per field was divided by the number of muscle fibers per field (n = 5 in each group). (D) VEGF, HGF and IGF synthesis in ischemic tissue determined by real-time PCR at day 7 after surgery following transplantation of Flk-1^+^ cells or PBS. VEGF, HGF or IGF mRNA levels were expressed relative to GAPDH mRNA levels (n = 5 in each group). N.S. = no significant difference between groups.

We examined whether young and old murine iPS cell-derived Flk-1^+^ cells formed vascular-like structures by themselves. Figure 2A shows representative photographs of cultured Flk-1^+^ cells from young and old murine iPS cells on a Matrigel matrix. The formation of network structures was successful, when Flk-1^+^ cells from both young and old murine iPS cells were cultured 3-dimensionally. Quantitative analyses of network formation showed no significant difference between young and old murine Flk-1^+^ cells (Figure 2A). We also assessed Flk-1^+^ cell incorporation by seeding HUVEC and young or old murine iPS cells on Matrigel (Figure 2B). Incorporation of Flk-1^+^ cells, derived from iPS cells from young and old mice into network structures, was confirmed. There is no difference in the number of Flk-1^+^ cell incorporation into HUVEC on Matrigel in between two groups (Figure 2B). Collectively, iPS cells derived from old mice differentiated into vascular cells with a time course and efficacy similar to those of iPS cells from young mice *in vitro*.

### Expansion and Senescence of Flk-1^+^ Cells Derived from iPS Cells from Young and Old Mice

We next assessed expansion and cellular senescence in Flk-1^+^ cells from young and old murine iPS cells. Figure 3A shows representative photographs of SA-β-Gal staining in undifferentiated and differentiated iPS cells. Under undifferentiated conditions, no expression of SA-β-Gal was detected in young and old murine iPS cells. Flk-1^+^ cells from iPS cells derived from young mice exhibited a robust growth rate, and less than 1% of these cells stained positive for SA-β-Gal for at least 8 passages (Figure 3B). In contrast, Flk-1^+^ cells from iPS cells derived from old mice grew very slowly, and 10–15% of these cells already stained positive for SA-β-Gal within 1–2 passages (Figure 3B). More than 20% of the cells expressed SA-β-Gal at passage 8, and then stopped proliferating (Figure 3B). Senescence associated genes such as ARF and p21 are contributed to the process of iPS reprogramming and senescence [16] Therefore, we assessed the mRNA levels of SIRT, ARF and p21 in undifferentiated and differentiated iPS cells. Under undifferentiated conditions, the mRNA levels of SIRT, ARF and p21 were similar in young and old murine iPS cells (Figure 3 C, D and E). However, SIRT mRNA levels in Flk-1^+^ cells from iPS cells derived from old mice were significantly lower than those from young mice in both phase of early and late passage (Figure 3 C). ARF and p21 levels in Flk-1^+^ cells from iPS cells derived from old mice were higher than those from young mice in both phase of early and late passage (Figure 3 D and E). Thus, Flk-1^+^ cells from old murine iPS cells show senescence in the early growth phase.

### Augmentation of Ischemia-induced Angiogenesis by Flk-1^+^ Cells Derived from iPS Cells from Young and old Mice

We examined whether implantation of Flk-1^+^ cells from iPS cells derived from young and old mice can augment ischemia-induced angiogenesis using a murine model of hindlimb ischemia *in vivo.* All mice survived the surgery and appeared healthy during the follow-up period. Body weight and blood pressure did not differ among the groups. Figure 4A shows representative LDBF images of hindlimb blood flow immediately after the ischemic surgery and at different time points thereafter. In control mice, hindlimb perfusion decreased precipitously after surgery, rose to 20–30% of that in the non-ischemic limb by day 3, and then increased to 40–50% of the non-ischemic limb value by day 14. A greater degree of blood perfusion was observed in the ischemic limbs of mice transplanted with Flk-1^+^ cells derived from young and old mice as compared to the control group (Figure 4B). It is noteworthy that augmentation of blood flow was accelerated by transplantation of Flk-1^+^ cells, and that the degrees of augmentation were similar in animals receiving cells derived from young and old mice.

To further investigate the extent of angiogenesis at the microcirculatory level, capillary density was also measured in histological sections harvested from the ischemic adductor muscle. Figure 4C shows a quantitative analysis revealing that, on postoperative day 14, transplantation of Flk-1^+^ cells derived from iPS cells from both young and old mice significantly increased capillary density in ischemic muscle as compared to controls. The capillary density after transplantation of old murine Flk-1^+^ cells was similar to that after transplantation of young murine Flk-1^+^ cells.

We next investigated whether transplantation of Flk-1^+^ cells stimulated the expression of angiogenic factors such as VEGF, HGF and IGF in ischemic hindlimb tissues. At postoperative day 7, VEGF, HGF and IGF mRNA levels were increased in mice transplanted either young or old murine Flk-1^+^ cells as compared to the control. The mRNA levels of VEGF, HGF and IGF after cell transplantation did not differ between young and old murine Flk-1^+^ cells (Figure 4D, E and F).

Furthermore, we examined whether implantation of Flk-1^+^ cells from iPS cells derived from young and old mice can augment ischemia-induced angiogenesis using different aged mice (18 to 20 weeks of age), because the reprogramming might be enhanced under the better environment such as injection into young mice. The degrees of augmentation were similar in mice, at the ages of 18 to 20 weeks, receiving cells derived from young and old mice (Figure S2A and B).

**Figure 5:**
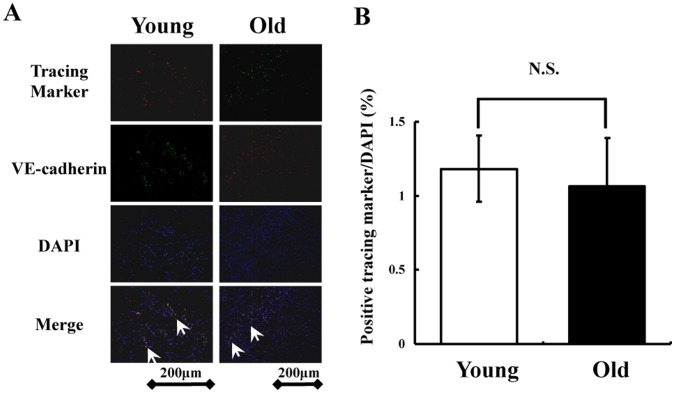
Tracking Flk-1^+^ cells during the chronic phase *in vivo*. (A) PKH26 labeled Flk-1^+^ cells from young iPS cells (red) and EGFP labeled Flk-1^+^ cells from old iPS cells (green) in ischemic muscle on postoperative day 21. Double fluorescence staining of VE-cadherin and labeled Flk-1^+^ cells in ischemic muscle. Co-localization is indicated by yellow in the merged images (magnification, ×200; bar indicates 200 µm). Total nuclei was identified by DAPI counterstaining (blue). (B) Quantitative analysis of the number of implanted Flk-1^+^ cells from young and old murine iPS cells in the chronic phase (n = 4 in each group).

Finally, we examined whether *in vivo* implanted Flk-1^+^ cells from young and old murine iPS cells can differentiate into endothelial cells in the chronic phase. PKH26 labeled Flk-1^+^ cells from young iPS cells (red) and EGFP labeled Flk-1^+^ cells from old iPS cells (green) were found in the ischemic area at postoperative day 21, and some of these cells seemed to be incorporated into VE-cadherin^+^ endothelial cells (Figure 5A). There were no significant differences in the proportion of these cells after cell transplantation between young and old murine Flk-1^+^ cells (Figure 5B). Furthermore, we detected no tumor formation in mice transplanted with Flk-1^+^ cells from young or older mice throughout the 40-day observation period (n = 3/each group, data not shown).

## Discussion

Major findings of the present study are as follows: (1) Flk-1^+^ cells derived from iPS cells obtained from old mice differentiated into mature vascular cells with a time course and efficacy similar to those of young murine iPS cells. (2) Flk-1^+^ cells from old murine iPS cells showed early cellular senescence. (3) The degree of revascularization with transplantation of Flk-1^+^ cells was similar between young and old murine iPS cells. (4) Implanted Flk-1^+^ cells derived from iPS cells obtained from young and old mice both differentiated into endothelial cells in the chronic phase *in vivo*.

With the proportion of people over age 60 years growing rapidly in industrial countries, the need for developing regenerative medicine strategies for the elderly population is a high priority [7]. However, lack of a fundamental understanding of the intrinsic changes that occur in cells during aging is one of the hurdles to the development of regenerative medicine strategies that will prove effective in older people. In experimental studies, a diminished responsiveness of tissue-specific stem and progenitor cells with advancing age leads to declining tissue regenerative capacity [17], [18], [19], [20]. Age-related declines in progenitor cell activity can be ameliorated by a “youthful” environment, but not an “elderly” environment [21]. In the clinical setting, iPS cells for personalized cell therapy require the establishment of iPS cell lines from relatively old autologous tissue, when we utilize iPS cells as a cell source for regenerative medicine. However, comparative angiogenic activities of iPS cells derived from young and old tissues have not been fully clarified. We established iPS cells from 21-month-old mice using bone marrow-derived myeloid cells [12]. In the present study, we compared angiogenic activities of iPS cells derived from young and old mice. Both young and old murine iPS cells differentiated into cardiovascular progenitor cells, showing equal capacity, and continued to differentiate into mature vascular cells.

We previously showed that direct local implantation of mouse iPS cell-derived Flk-1^+^ cells augmented VEGF expression in ischemic tissues [11]. In the present study, we confirmed that VEGF, HGF and IGF mRNA levels in ischemic tissues were increased in mice transplanted either young or old murine Flk-1^+^ cells. Transplanted Flk-1^+^ cells from both young and old murine iPS cells were engrafted into the muscles with a limited area, and some cells were still present at 3 weeks after the transplantation. However, the proportions of Flk-1^+^ cells from both young and old murine at 3 weeks after the transplantation were very low. Collectively, major mechanisms of iPS cell therapies are most likely mediated through angiogenic cytokines released from host skeletal myoblasts rather than by a direct differentiation of transplanted cells into mature endothelial cells.

A recent study using heterochromic parabiotic parings between young and old mice showed that circulating systemic factors from a young mouse could restore muscle tissue regeneration after injury in aged mice [21]. Circulating factors from young mice also increased the engraftment potential of endogenous hematopoietic stem cells in the old mice in the parabiosis model. Thus, for a stem cell transplantation-based strategy, one important consideration is the age of the donor tissue and the recipient environment. In the present study, the degree of augmentation of revascularization after ischemic injury with transplantation of Flk-1^+^ cells was similar for young and old murine iPS cells. Recently, it was reported that telomerase has a prolonged half-life in iPS cells [22], whereas the progressive shortening of telomeres is an intrinsic change that ultimately restricts the number of divisions [23]. Collectively, iPS cells might resolve intrinsic aging problems such as telomere shortening, and even iPS cells derived from old mice retain their capacity to become vascular progenitor cells.

It was recently shown that human iPS cells established from adults are capable of differentiating into hemangioblasts, but efficiency is dramatically decreased and early senescence was observed [24]. In the current study, Flk-1^+^ cells derived from iPS cells obtained from old mice showed senescence in the early growth phase. Chin et al [25] conducted a genome-wide study to compare iPS cells with ES cells. Their comparison of expression patterns indicated that in the early passage iPS cell lines are incompletely reset to an ES cell-like expression pattern, and even late passage differences between ES cells and iPS cells persist and reflect an imperfect resetting of somatic cell expression to an ES cell-like state. Thus, these differences in gene expression may lead to excessive senescence in iPS cells derived from old mice. It is necessary to carefully monitor the possibility of early senescence, when we utilize iPS cells from elderly subjects. Future studies will be required to explore the factors modifying old iPS cells and their role in possibly enhancing the success of cell therapies in elderly patients.

Previously, we reported the differentiation capacities of young and old murine iPS cells into myeloid lineage [12]. The two iPS cell lines had similar biological characteristics and showed similar patterns in differentiation into myeloid lineage as well as vascular cell. However, the origins of donor cells used for inducing iPS from young and old mice were different (embryonic fibroblasts vs. dendritic cells). Epigenetic memories may be specifically related to their tissue origins beyond the age differences. Therefore, detailed studies using various experimental models are required to better understand the effect of aging on the differentiation capacity of iPS cells.

This study has several limitations. First, it is unclear how the secretion of angiogenic cytokines is induced and maintained in skeletal myoblasts by the cell implantation. We assessed the expression of VEGF in Flk-1^+^ cells *in vitro* using Proteome Profiler array. Little or no expression of VEGF was detected in iPS cell-derived Flk-1^+^ cells (data not shown). Thus, angiogenic cytokines such as VEGF might not release from implanted iPS cell-derived Flk-1^+^ cells. Detailed biochemical studies are required to understand the precise mechanisms of the secretion of angiogenic cytokines by the iPS cell therapies. Second, the relationship between endogenous endothelial progenitor cells (EPCs) and Flk-1^+^ cells from iPS cell has not been clarified. The induction of EPCs into the ischemic limbs might be accelerated by the implantation of iPS cell-derived Flk-1^+^ cells.

In conclusion, mouse iPS cell-derived Flk-1^+^ cells differentiated into vascular cells, and regulated angiogenic vascular responses both *in vitro* and *in vivo*. These properties of old murine iPS cells are largely comparable to those of iPS cells from young mice, which suggests the functionality of the generated iPS cells themselves to be unaffected by aging. Our current results indicate that iPS cells are potentially good alternatives to bone marrow or circulating progenitor cells for angiogenesis induction.

## Supporting Information

Figure S1 **Time course of differentiating Flk-1 positive cells.** (A) The expression of Flk-1 peaked at Day7.5 after the completion of differentiation. The time course and average ratio of emerging Flk-1^+^ cells were similar for old (BM21) and young (MEF) iPS cells. N.S. indicates no significant difference. (B) Purification of Flk-1^+^ cells from iPS cells. FACS analysis of pre and post MACS-sorted Flk-1^+^ cells at day 7.5. More than 90% of enriched cells were positive for Flk-1. Old iPS cells were consistently positive for GFP.
(TIF)

Figure S2 **Effects of cell transplantation on blood flow recovery in the ischemic hindlimb of aged mice.** (A) Representative LDBF images. A low perfusion signal (dark blue) was observed in the ischemic left hindlimb of control mice (PBS), whereas high perfusion signals (white to red) were detected in the ischemic left hindlimb of mice transplanted with Flk-1^+^ cells derived from young and old mice (2×10^5^ cells) on postoperative days 3, 7 and 14. (B) Quantitative analysis of the ischemic to non-ischemic limb LDBF ratio on pre- (Day-1) and postoperative days 0, 3, 7 and 14 (Control: n = 8, Young: n = 4, Old: n = 4). *p<0.05 for mice injected with Flk1^+^ cells (2×10^5^) vs. control mice.
(TIF)

## References

[1] Tateishi-Yuyama E, Matsubara H, Murohara T, Ikeda U, Shintani S (2002). Therapeutic angiogenesis for patients with limb ischaemia by autologous transplantation of bone-marrow cells: a pilot study and a randomised controlled trial.. Lancet.

[2] Kajiguchi M, Kondo T, Izawa H, Kobayashi M, Yamamoto K (2007). Safety and efficacy of autologous progenitor cell transplantation for therapeutic angiogenesis in patients with critical limb ischemia.. Circ J.

[3] Devanesan AJ, Laughlan KA, Girn HR, Homer-Vanniasinkam S (2009). Endothelial progenitor cells as a therapeutic option in peripheral arterial disease.. Eur J Vasc Endovasc Surg.

[4] Kumar AH, Caplice NM (2010). Clinical potential of adult vascular progenitor cells.. Arterioscler Thromb Vasc Biol.

[5] Mund JA, Ingram DA, Yoder MC, Case J (2009). Endothelial progenitor cells and cardiovascular cell-based therapies.. Cytotherapy.

[6] Piepoli MF (2009). Transplantation of progenitor cells and regeneration of damaged myocardium: more facts or doubts? Insights from experimental and clinical studies.. J Cardiovasc Med (Hagerstown).

[7] Piccin D, Morshead CM (2010). Potential and pitfalls of stem cell therapy in old age.. Dis Model Mech.

[8] Takahashi K, Yamanaka S (2006). Induction of pluripotent stem cells from mouse embryonic and adult fibroblast cultures by defined factors.. Cell.

[9] Narazaki G, Uosaki H, Teranishi M, Okita K, Kim B (2008). Directed and systematic differentiation of cardiovascular cells from mouse induced pluripotent stem cells.. Circulation.

[10] Taura D, Sone M, Homma K, Oyamada N, Takahashi K (2009). Induction and isolation of vascular cells from human induced pluripotent stem cells–brief report.. Arterioscler Thromb Vasc Biol.

[11] Suzuki H, Shibata R, Kito T, Ishii M, Li P (2010). Therapeutic angiogenesis by transplantation of induced pluripotent stem cell-derived Flk-1 positive cells.. BMC Cell Biol.

[12] Zhao Cheng SI, Hengyi Xiao, Haruhiko Suzuki, Yayoi Okawa, Toyoaki Murohara (2010). Establishment of induced pluripotent stem cells from aged mice using bone marrow-derived myeloid cells.. Journal of Molecular Cell Biology.

[13] Murohara T, Asahara T, Silver M, Bauters C, Masuda H (1998). Nitric oxide synthase modulates angiogenesis in response to tissue ischemia.. J Clin Invest.

[14] Couffinhal T, Silver M, Zheng L, Kearney M, Witzenbichler B (1998). Mouse model of angiogenesis.. Am J Pathol.

[15] Shibata R, Ouchi N, Kihara S, Sato K, Funahashi T (2004). Adiponectin stimulates angiogenesis in response to tissue ischemia through stimulation of amp-activated protein kinase signaling.. J Biol Chem.

[16] Banito A, Rashid ST, Acosta JC, Li S, Pereira CF (2009). Senescence impairs successful reprogramming to pluripotent stem cells.. Genes Dev.

[17] Conboy IM, Conboy MJ, Smythe GM, Rando TA (2003). Notch-mediated restoration of regenerative potential to aged muscle.. Science.

[18] Fuller J (2002). Hematopoietic stem cells and aging.. Sci Aging Knowledge Environ.

[19] Sigal SH, Brill S, Fiorino AS, Reid LM (1992). The liver as a stem cell and lineage system.. Am J Physiol.

[20] Kuhn HG, Dickinson-Anson H, Gage FH (1996). Neurogenesis in the dentate gyrus of the adult rat: age-related decrease of neuronal progenitor proliferation.. J Neurosci.

[21] Conboy IM, Conboy MJ, Wagers AJ, Girma ER, Weissman IL (2005). Rejuvenation of aged progenitor cells by exposure to a young systemic environment.. Nature.

[22] Agarwal S, Loh YH, McLoughlin EM, Huang J, Park IH (2010). Telomere elongation in induced pluripotent stem cells from dyskeratosis congenita patients.. Nature.

[23] Gilson E, Géli V (2007). How telomeres are replicated.. Nat Rev Mol Cell Biol.

[24] Feng Q, Lu SJ, Klimanskaya I, Gomes I, Kim D (2010). Hemangioblastic derivatives from human induced pluripotent stem cells exhibit limited expansion and early senescence.. Stem Cells.

[25] Chin MH, Mason MJ, Xie W, Volinia S, Singer M (2009). Induced pluripotent stem cells and embryonic stem cells are distinguished by gene expression signatures.. Cell Stem Cell.

