# Supplementary figures and images for: Comparative Angiogenic Activities of Induced Pluripotent Stem Cells Derived from Young and Old Mice
Source: PLoS One. 2012 Jun 27;7(6):e39562. doi: 10.1371/journal.pone.0039562 (PMC3384644; doi:10.1371/journal.pone.0039562)

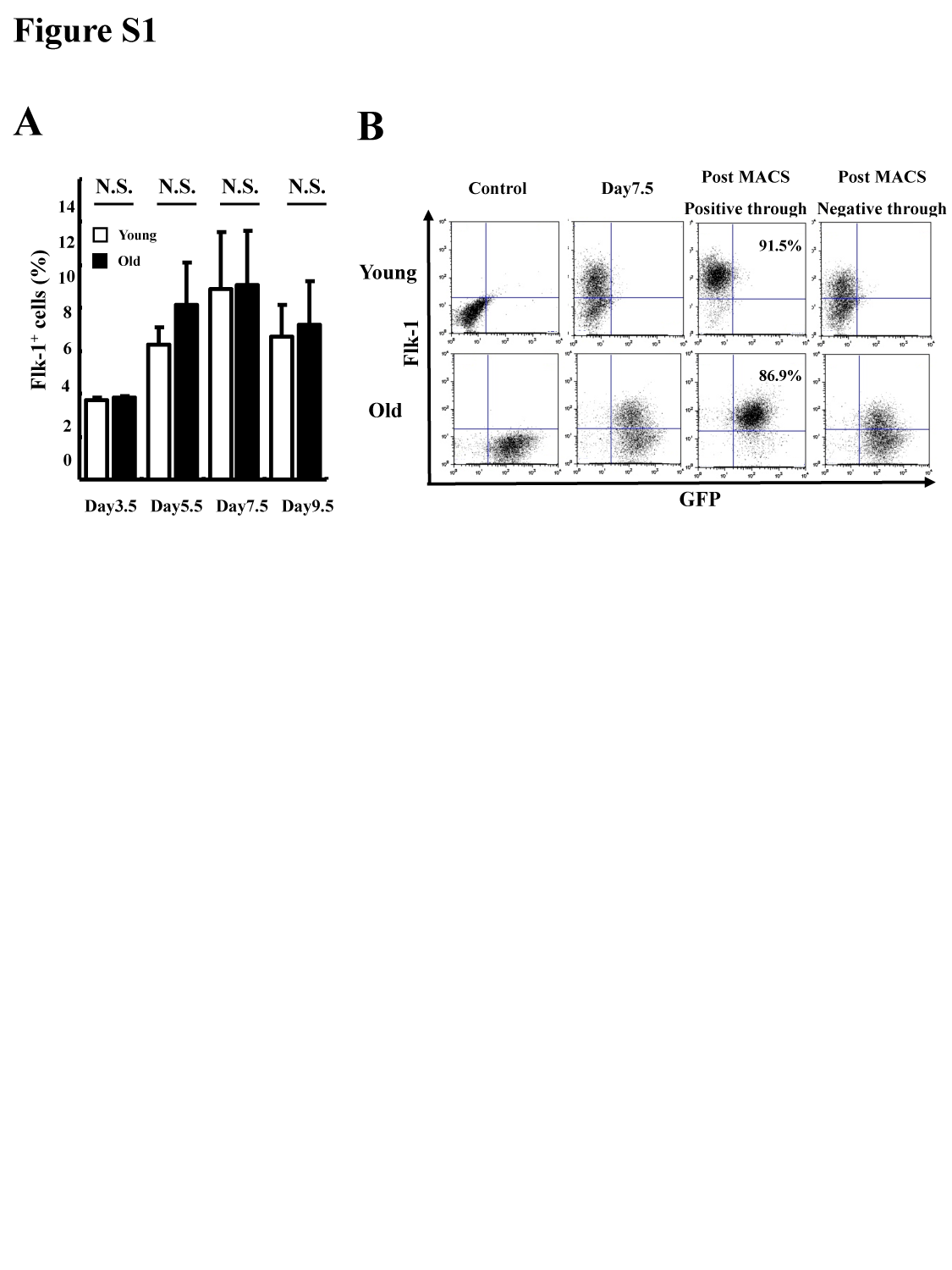

Supplement: Figure S1 — Time course of differentiating Flk-1 positive cells. (A) The expression of Flk-1 peaked at Day7.5 after the completion of differentiation. The time course and average ratio of emerging Flk-1+ cells were similar for old (BM21) and young (MEF) iPS cells. N.S. indicates no significant difference. (B) Purification of Flk-1+ cells from iPS cells. FACS analysis of pre and post MACS-sorted Flk-1+ cells at day 7.5. More than 90% of enriched cells were positive for Flk-1. Old iPS cells were consistently positive for GFP. (TIF) [file pone.0039562.s001.tif]

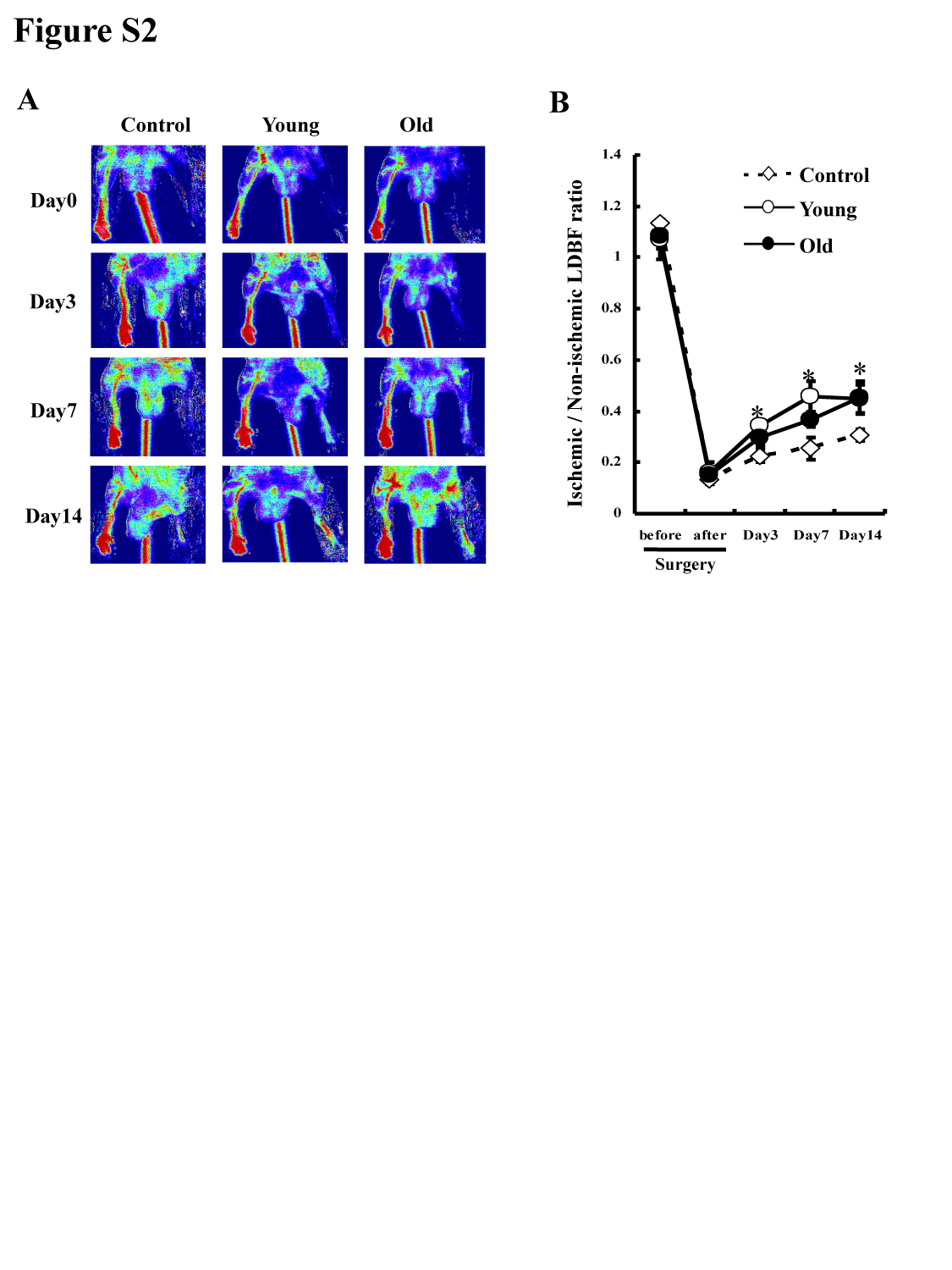

Supplement: Figure S2 — Effects of cell transplantation on blood flow recovery in the ischemic hindlimb of aged mice. (A) Representative LDBF images. A low perfusion signal (dark blue) was observed in the ischemic left hindlimb of control mice (PBS), whereas high perfusion signals (white to red) were detected in the ischemic left hindlimb of mice transplanted with Flk-1+ cells derived from young and old mice (2×10^5^ cells) on postoperative days 3, 7 and 14. (B) Quantitative analysis of the ischemic to non-ischemic limb LDBF ratio on pre- (Day-1) and postoperative days 0, 3, 7 and 14 (Control: n = 8, Young: n = 4, Old: n = 4). *p<0.05 for mice injected with Flk1+ cells (2×10^5^) vs. control mice. (TIF) [file pone.0039562.s002.tif]
